# Quality Screening of Incorrectly Folded Soluble Aggregates from Functional Recombinant Proteins

**DOI:** 10.3390/ijms20040907

**Published:** 2019-02-19

**Authors:** Soon Bin Kwon, Ji Eun Yu, Jihoon Kim, Hana Oh, Chan Park, Jinhee Lee, Baik L. Seong

**Affiliations:** Department of Biotechnology, College of Life Science and Biotechnology, Yonsei University, Seoul 03722, Korea; yunbin829@gmail.com (S.B.K.); 4-season-love@hanmail.net (J.E.Y.); wlgns2568@naver.com (J.K.); ohanah@naver.com (H.O.); justies@naver.com (C.P.); glory-jjin@hanmail.net (J.L.)

**Keywords:** recombinant protein, soluble aggregates, cell lysis, sonication, mild lysis

## Abstract

Solubility is the prime criterion for determining the quality of recombinant proteins, yet it often fails to represent functional activity due to the involvement of non-functional, misfolded, soluble aggregates, which compromise the quality of recombinant proteins. However, guidelines for the quality assessment of soluble proteins have neither been proposed nor rigorously validated experimentally. Using the aggregation-prone enhanced green-fluorescent protein (EGFP) folding reporter system, we evaluated the folding status of recombinant proteins by employing the commonly used sonication and mild lysis of recombinant host cells. We showed that the differential screening of solubility and folding competence is crucial for improving the quality of recombinant proteins without sacrificing their yield. These results highlight the importance of screening out incorrectly folded soluble aggregates at the initial purification step to ensure the functional quality of recombinant proteins.

## 1. Introduction

Bacterial hosts, in particular *Escherichia coli*, have been favored for the recombinant expression of heterologous proteins as the procedure is inexpensive, fast, simple, and easy to scale-up. Nevertheless, the production of properly folded heterologous proteins in *E. coli* is still difficult, necessitating optimal conditions for the expression and purification of the protein of interest. The cytoplasm of *E. coli* often fails to provide the optimal environment for the folding of recombinant proteins; therefore, various approaches have been used to overcome this inherent problem [1]. These approaches include: (1) co-expression with molecular chaperones or specific binding partners that may help to fold target proteins [2,3,4,5], and (2) fusion with highly soluble proteins, such as Glutathione S-transferase(GST), Maltose-binding protein(MBP), N utilization substance protein A(NusA) [6], DnaK [7,8], Small ubiquitin-like modifier(SUMO) [9], or Lysyl tRNA synthetase(LysRS) [10], to enhance the solubility of target proteins [11]. Alternatively, approaches based on the chaperone function of RNAs (chaperna; chaperone + RNA) [12], co-expression with a specific RNA ligand [4,5], or fusion with an RNA-interaction domain (RID) [13,14,15] greatly enhances the solubility and the overall yield of soluble proteins.

Although there have been many advances in terms of enhancing the solubility of proteins, solubility itself cannot guarantee the adequate biological function of the protein of interest, which is primarily due to the formation of soluble aggregates [16,17,18]. Soluble aggregates are soluble oligomers that form micelle-like structures, which are usually biologically inert. Although fusion with highly soluble partners or co-expression with molecular chaperones may greatly enhance their overall solubility, it has long been observed that these proteins often remain biologically inactive. Further examination of their physico-chemical properties frequently reveals that these proteins are present predominantly as aggregates rather than in their mono-dispersed forms [18]. Such negative results are regularly observed by researchers, but are not usually shared in the public domain.

Thus, novel methods are required to identify or screen out the soluble aggregates and soluble-but-inactive proteins from the correctly folded proteins. This is particularly important to ensure the quality of purified proteins for detailed analyses of the biological function of proteins. Even though screening out soluble-but-inactive proteins remains a challenge, soluble aggregates can be separated based on their physical characteristics. In particular, dynamic light scattering (DLS) can be used to identify soluble aggregates; however, it requires highly purified protein samples without other cellular components. Size exclusion chromatography (SEC) is used to separate soluble aggregates from mono-dispersed proteins in the void volume, but again this procedure requires prior purification steps. Moreover, SEC is unsuitable for large-scale industrial production due to the limited capacity of columns. Thus, a simple and practical procedure to screen out soluble aggregates is required prior to downstream purification steps.

Herein, we used enhanced green-fluorescent protein (EGFP) as a reporter protein for soluble expression and functional assays. Previously, we observed that the RNA-interaction domain (RID) from human LysRS (hRID) can be used as a solubility enhancer for aggregation-prone proteins [13,14], whilst the K23A-K27A mutation (hRID(2m)), which abolishes RNA affinity, was shown to induce soluble aggregates [13,14]. Here, we fused hRID(2m) to the N-terminal of EGFP to induce the formation of soluble aggregates. Using this model protein prone to soluble aggregation, and employing commonly used cell extraction methods from the *E. coli* recombinant host, we showed that the differential screening of solubility and folding competence is a prerequisite for improving the quality of recombinant proteins in subsequent purification processes.

## 2. Results

### 2.1. Sonication Negatively Affects Protein Quality

**Figure 1:**
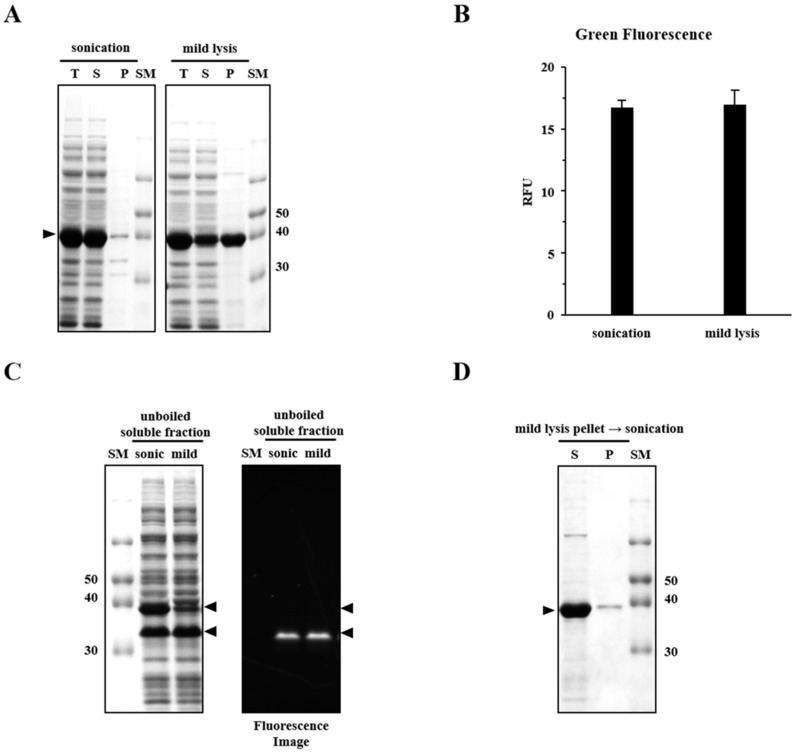
Sonication decreases hRID(2m)-EGFP quality by increasing incompletely folded hRID(2m)-EGFP. (**A**) Expression of hRID(2m)-EGFP in *E. coli*. The culture was incubated at 37 °C with IPTG, and the cell extract made either by sonication (left panel) or mild lysis (right panel) was analyzed by SDS-PAGE. (**B**) Fluorescence of hRID(2m)-EGFP in the soluble fractions obtained by sonication or mild lysis was measured using a fluorescence spectrometer. Vertical bars represent the mean ± S.D. (**C**) hRID(2m)-EGFP in the soluble fraction was analyzed by SDS-PAGE without boiling. Both a Coomassie-stained image (left panel) and a fluorescence image (right panel) are shown. (**D**) The mild lysis pellet fraction was suspended in a buffer, sonicated, and divided into the supernatant and pellet by centrifugation. SM: size marker, T: total, S: soluble, P: pellet, RFU: relative fluorescence units.

We compared two different methods of cell disruption: sonication (a physical method) and mild lysis (B-PER; a chemical method). Since EGFP is expressed predominantly in its insoluble form in *E. coli* under harsh conditions (overexpression at 37 °C), hRID(2m)-fusion was used to regulate the formation of soluble aggregates. Previously, hRID (N-terminal appended domain of human lysyl tRNA synthetase) was shown to increase the solubility and folding of fused proteins, while hRID(2m) was shown to increase the abundance of soluble aggregates [13,14,15]. The solubility of hRID(2m)-EGFP was analyzed using sodium dodecyl sulfate-polyacrylamide gel electrophoresis (SDS-PAGE) after dividing the samples into total (T), soluble (S), and pellet (P) fractions by centrifugation (Figure 1A). The solubility of the protein obtained was >95% after sonication and <50% after B-PER treatment. This result was unexpected, considering that local temperature increase and free radical formation by sonication are known to promote protein aggregation [19,20]. Interestingly, there was no significant difference in the relative fluorescence units (RFUs) despite there being a higher amount of soluble protein in the sonicated sample (Figure 1B). We, therefore, suspected that the folding quality of EGFP in the soluble fraction after sonication was poorer than after mild lysis, as suggested by SDS-PAGE analysis (Figure 1C). A chromophore is formed when EGFP is properly folded [21]. This chromophore-mature conformation has been reported to be quite stable, retaining its 3D structure even in the presence of low concentrations of detergents such as SDS; however, the fluorescence disappears after boiling [22,23]. Without boiling, the migration velocity of EGFP in SDS-PAGE is dependent on the formation of a mature chromophore [23]. As shown in Figure 1C, sonication resulted in two different bands, one with retarded migration (~39 kDa), which was reproducibly observed in normal SDS-PAGE with heat treatment prior to gel loading (Figure 1A), and the other with faster migration (~32 kDa). Notably, the fast migrating lower band was fluorescent, confirming the formation of a mature chromophore, whereas the other band was not fluorescent (Figure 1C). A remarkable difference was observed between the two different EGFP preparations; while the lower band remained the same, the upper band was greatly enriched after sonication. After mild lysis, the pellet fraction appeared to become soluble after further sonication, as >95% of the proteins in the pellet fraction were transferred to the soluble fraction after sonication (Figure 1D). These results suggest that: (1) the EGFP fraction that successfully folded via chromophore formation is stably released into the soluble fraction after mild lysis, (2) incorrectly folded EGFPs are still associated with bacterial cell components that resist elution by mild lysis, and (3) the physical disruption of recombinant cells by sonication is strong enough to dissociate the incompletely folded oligomer complex into smaller sized oligomers that survive precipitation by centrifugation.

**Figure 2:**
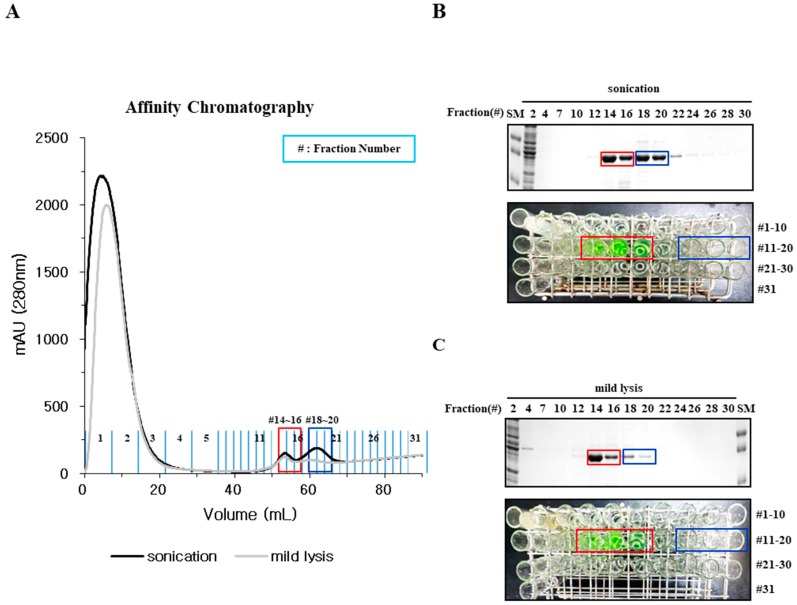
Separation of competently and incorrectly folded hRID(2m)-EGFP by nickel (Ni)-affinity chromatography. (**A**) Soluble hRID(2m)-EGFP obtained by sonication or mild lysis was purified using Ni-affinity chromatography. Black line: sonication; grey line: mild lysis. (**B**,**C**) SDS-PAGE analysis of (**B**) sonication and (**C**) mild lysis fractions. Lower panels are photographs; upper panels represent SDS-PAGE of fractions in 2A. Red box: fractions #14-16; Blue box; fractions #18-20 in 2A.

The folding behavior of EGFP was analyzed during subsequent chromatographic purification steps. The cell extract from a 500 mL *E. coli* culture was loaded onto a Ni-NTA column (GE healthcare). EGFP was eluted in two distinctive peaks, where sonication resulted in two peaks with a slight enrichment of the later fraction (blue box) and mild lysis enriched the earlier fraction (red box) (Figure 2A). The purified proteins from both fractions were a similar size to those observed by SDS-PAGE, but again only the earlier fraction was fluorescent (Figure 2B,C). These results confirm and further extend the observations shown in Figure 1 that mild lysis, but not sonication, was able to enrich the levels of properly folded EGFP protein. These results also highlight the importance of screening properly folded proteins prior to downstream chromatographic purification steps.

### 2.2. Sonication Increases the Level of Incorrectly Folded Soluble Aggregates

**Figure 3:**
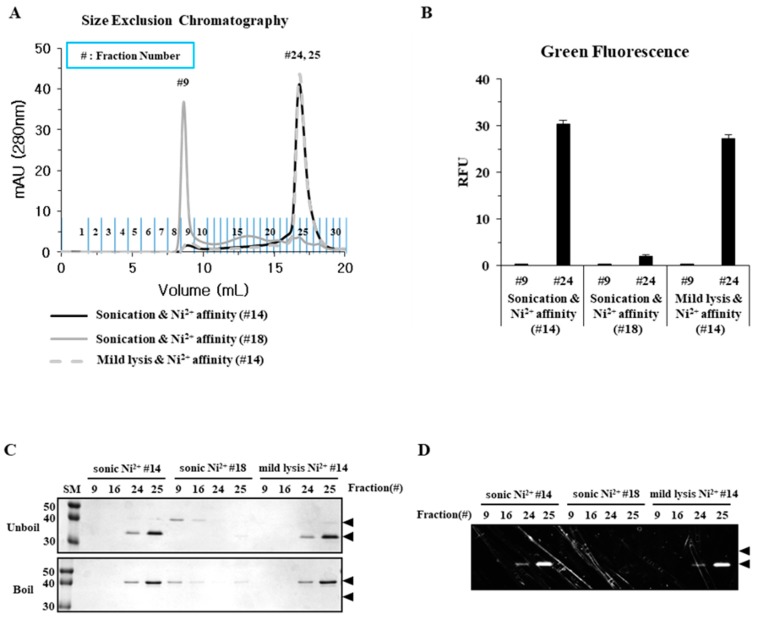
Sonication increases the level of incorrectly folded soluble aggregates and decreases hRID(2m)-EGFP quality. (**A**) Size exclusion chromatography (SEC) of purified hRID(2m)-EGFP fractions from Ni-affinity chromatography (Figure 2). Fractions #14 (fluorescent) and #18 (non-fluorescent) from Ni-affinity chromatography of the protein obtained by sonication were compared (black and grey lines, respectively). Fraction #14 from Ni-affinity chromatography of the protein obtained by mild lysis (grey dotted line). (**B**) Fluorescence of each SEC fraction. (**C**) SDS-PAGE analysis of each fraction with or without boiling prior to gel loading. (**D**) Fluorescence image of SDS-PAGE gel (C, upper panel) obtained using a gel documentation system.

The composition of the fractions purified by Ni-affinity chromatography was analyzed by size exclusion chromatography (SEC). We examined three different fractions: fractions 14 and 18 obtained by sonication (Figure 2B, red and blue boxes, respectively), and fraction 14 obtained by mild lysis (Figure 2C, red box). Fraction 18 was eluted predominantly in the void volume (SEC fraction 9) and very little, if any, was eluted in the later fractions (24 and 25; Figure 3A). Elution in the void volume suggests that the protein is in the form of soluble aggregates of high molecular weight (MW). None of the corresponding fraction 9 from any of the three different preparations was fluorescent (Figure 3B). In contrast, fraction 24 was fluorescent after both sonication and mild lysis, indicating proper folding. The MW of SEC fractions 24 and 25 was estimated to be approximately 100 kDa, corresponding with the dimeric form of hRID(2m)-EGFP (Figure 3A). When SDS-PAGE was performed without boiling, SEC fractions 24 and 25 exhibited faster migration after both mild lysis and sonication (Figure 3C). In contrast, the migration velocity of SEC fraction 9 did not change after boiling, consistent with the results presented in Figure 1C. Fractions 24 and 25 were fluorescent, but fraction 9 was not (Figure 3D), suggesting that the soluble aggregates released after sonication are incorrectly folded proteins.

### 2.3. Particle Size is a Determining Factor for the Solubilization of Incorrectly Folded Aggregates

**Figure 4:**
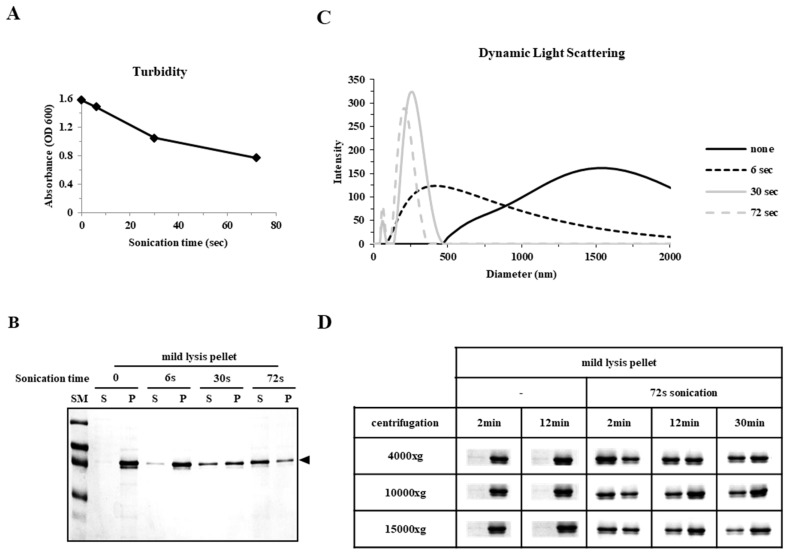
Solubilization of aggregated hRID(2m)-EGFP due to reduced particle size after sonication. (**A**) Time-dependent decrease in turbidity (OD 600 nm) by sonication. (**B**) Solubilization of mild lysis pellet fraction after sonication as analyzed by SDS-PAGE. (**C**) The particle size distribution of each sample in (**A**) by dynamic light scattering. (**D**) Monitoring of solubility change by SDS-PAGE after centrifugation at different forces for different lengths of time.

We analyzed the relationship between the physical characteristics and apparent solubility of incorrectly folded aggregates. The mild lysis pellet fraction was subjected to sonication, with turbidity gradually decreasing in a time-dependent manner (Figure 4A) alongside an increase in the soluble fraction when analyzed by SDS-PAGE after centrifugation (Figure 4B). The size distribution of aggregates was monitored by dynamic light scattering (DLS), with the average size gradually decreasing with prolonged sonication at 370, 260, and 220 nm, for 6, 30, and 72 s, respectively, when compared to 1500 nm without sonication (Figure 4C). These results showed that sonication could split pellet proteins into smaller, soluble aggregates that resisted precipitation by centrifugation. Therefore, we examined the effect of centrifugal force on the degree of protein precipitation. The mild lysis pellet fraction was suspended in buffer, sonicated (72 s), centrifuged at rotor speeds corresponding to 4000, 10,000, and 15,000× *g*, and analyzed by SDS-PAGE (Figure 4D). Samples that were not sonicated remained as a pellet regardless of centrifugal force, confirming that large aggregates (~1500 nm in DLS; Figure 4C) precipitate easily even at a low centrifugal force. Prolonged sonication (72 s) greatly increased the soluble fraction after centrifugation, however, the soluble fraction gradually decreased with increasing centrifugal force in a time dependent manner (Figure 4D). These results suggest that mild lysis effectively screened out non-functional incorrectly folded protein at the start of the purification process.

### 2.4. Mild Lysis Results in Better Recombinant RS-SPARC Protein Quality While Sonication Results in High Soluble Aggregate Contamination

**Figure 5:**
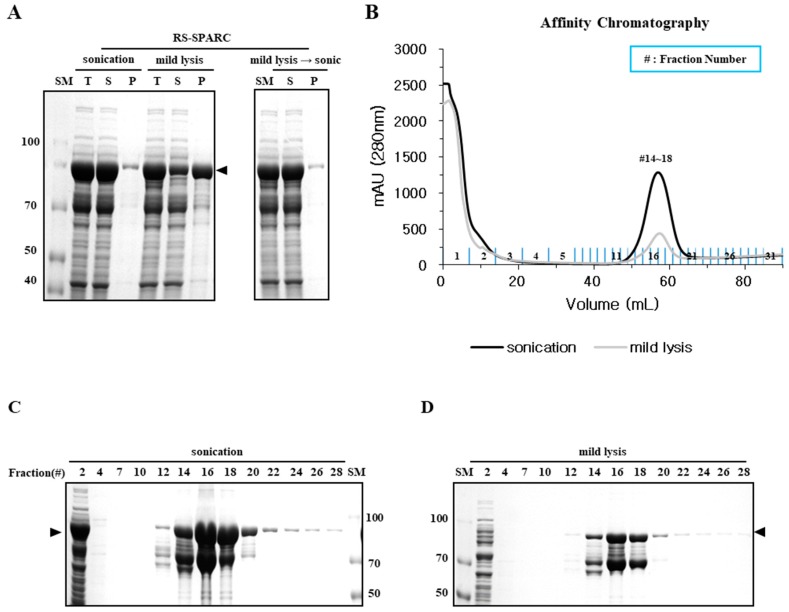
High quality RS-SPARC was purified by mild lysis. (**A**) Expression of RS-SPARC in *E. coli*. The culture was incubated at 25 °C with IPTG and the solubility of cell extracts was analyzed by SDS-PAGE. Comparison of sonication and mild lysis (left panel). The lysate obtained by mild lysis was sonicated before SDS-PAGE analysis (right panel). (**B**) Soluble fractions obtained by sonication and mild lysis were purified using Ni-affinity chromatography. (**C**,**D**) SDS-PAGE analysis of the fractions from B. (**C**) and (**D**) represent samples after sonication and mild lysis, respectively.

We also investigated other recombinant proteins known to form soluble aggregates. Previously, we observed that the *Clonorchis sinensis* SPARC protein is refractory to soluble expression in *E. coli*. To prevent the formation of inclusion bodies, the protein was expressed by fusion with LysRS, a potent soluble carrier protein [10]. The RS-SPARC fusion protein predominantly remained soluble (>95%) after sonication, however, the soluble yield was lower (~25%) after mild lysis (Figure 5A). Most of the mild lysis protein pellet was transferred to the soluble fraction (>95%) after sonication, equivalent to the soluble yield obtained from bacterial lysates after sonication. However, the physico-chemical properties of the soluble fraction were markedly different in a simple Ni-affinity chromatography. Higher yield was noted for sonicated samples in the eluted fraction, yet a significant portion was present in the flow-through (FT) fractions (fraction #1–3) that did not bind the Ni-affinity matrix (Figure 5C). This failure to bind the Ni-affinity column was probably due to shielding of the affinity tag (hexa His peptide at the fusion junction) by oligomeric aggregation. In contrast, despite having a lower yield than sonication (Figure 5B), the recombinant RS-SPARC prepared by mild lysis predominantly bound the Ni-affinity matrix, with very little, if any, left in the FT fractions (Figure 5D). It is likely that exposure of the His-tag enabled binding to the affinity column, reflecting a proper folding of expressed protein. The smaller band (~70kDa), co-purified with the RS-SPARC fusion protein (~90 kDa, arrow), was probably due to proteolytic cleavage near the fusion junction [24].

**Figure 6:**
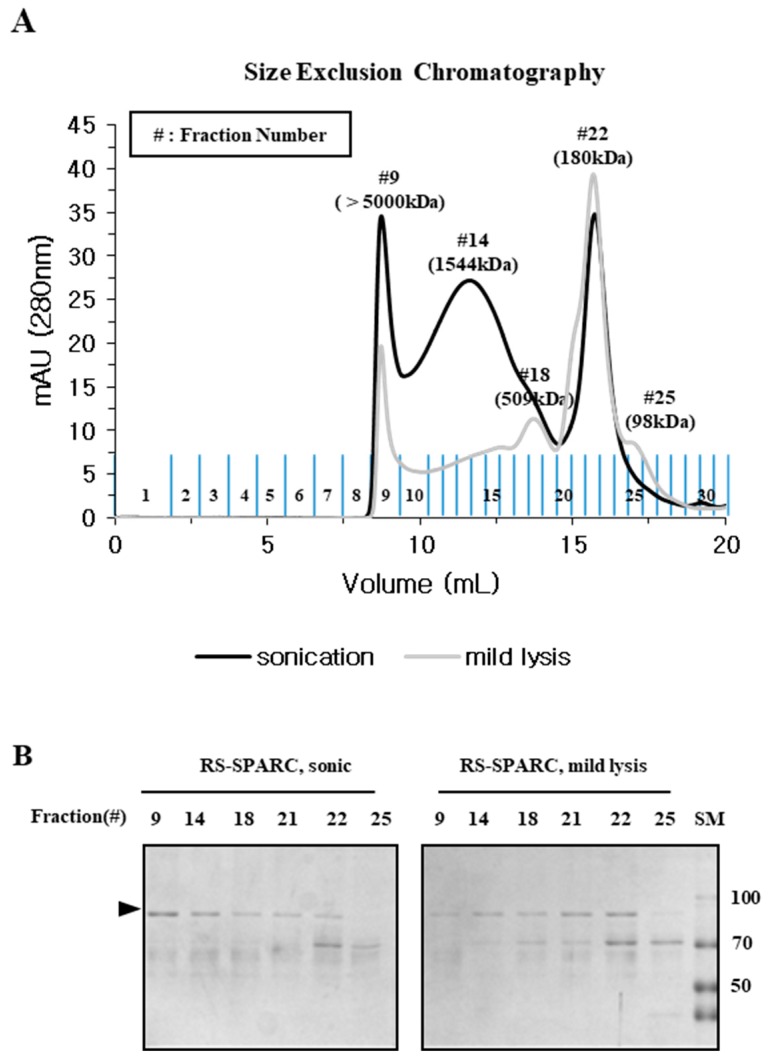
Mild lysis increases quality of RS-SPARC, while sonication increases incorrectly folded soluble RS-SPARC aggregates. (**A**) The Ni-affinity chromatography fractions (Figure 5B) were analyzed by SEC. Black line: SEC of Ni-affinity purified protein after sonication. Grey line: SEC of Ni-affinity purified protein after mild lysis. (**B**) The eluted SEC fractions were analyzed by SDS-PAGE.

The protein purified by Ni-affinity was analyzed by SEC. Properly folded fusion proteins were expected to form dimers due to the dimeric conformation of the LysRS protein [25]. The protein obtained by mild lysis was predominantly present as a dimer (~180 kDa), whereas the sample prepared by sonication consisted largely of oligomeric units (fraction #14, 1544 kDa) and void volume (fraction #9, >5000 kDa) in addition to the expected dimers (Figure 6A). The balance between dimers and multimeric oligomers differed significantly in the two samples when examined by SDS-PAGE (Figure 6B). Our results suggest that the presence of soluble oligomeric aggregates was a general phenomenon in the recombinant expression of target proteins, and further highlights the importance of screening the physico-chemical quality of soluble proteins at the beginning of purification.

## 3. Discussion

The recombinant expression of target proteins in bacterial hosts has long been hindered by the formation of insoluble inclusion bodies. As solubility is an initial criterion for determining the quality of recombinant proteins, various approaches have been used to increase their solubility [2,6,10,11]. However, guidelines for the quality assessment of soluble proteins have neither been proposed nor critically validated experimentally. Here, we employed the popular cell-lysis methods of sonication and mild lysis to examine the folding status of soluble proteins. EGFP was used as the folding reporter, owing to its distinct functional (with chromophores) and non-functional (without chromophores) forms, which can be identified by fluorescence and physico-chemical behavior in well-adapted gel electrophoresis (Figure 1C and Figure 3C) [23]. Here, soluble aggregates of EGFP were intentionally formed using an RNA-binding defective solubility enhancer [14,26].

The physical properties, primarily solubility after centrifugation and the status of mono-dispersity/oligomerization determined by SEC, were compared with the functional (fluorescence) and biochemical properties represented by different migration velocities in SDS-PAGE, which were dependent on chromophore formation. Mild lysis in a detergent buffer does not increase the local temperature, or generate free radicals that induce protein aggregation as sonication does [19,20]; however, we found that the solubility of sonicated samples was far better than that of mildly lysed samples (Figure 1 and Figure 5). Despite a higher soluble fraction yield after sonication, the level of functional proteins (as quantified by fluorescence and gel mobility) was similar to that obtained after mild lysis (Figure 1). Purification analysis showed that the functional form of EGFP was enriched in the soluble fractions from mild lysis (Figure 2). Therefore, the physical disruption of bacterial cells by sonication lowered the ratio of functional proteins by increasing the proportion of non-functional aggregates within the soluble fraction. It is likely that the physical energy of sonication is strong enough to fragment non-functional aggregates into smaller aggregates, which resist precipitation by centrifugation (Figure 3 and Figure 4). In contrast, despite initially having a lower soluble fraction yield, mild lysis led to the enrichment of properly folded proteins. Parallel experiments using SPARC, the biochemical function of which is still uncharacterized, supported these observations; sonication resulted in the enrichment of oligomeric proteins that failed to bind the affinity resin (Figure 5 and Figure 6).

The findings of this study support the use of mild lysis over sonication for the initial screening of soluble fractions by eliminating non-functional soluble aggregates. Mild lysis is much simpler than other methods, such as DLS or SEC, which are typically used in subsequent purification steps and require highly purified protein samples. With respect to volume requirement, DLS usually requires a few milliliters of purified samples for analysis. SEC has an upper limit in loading volume, so SEC is not recommended for large-scale purification. The physical limitations of these biophysical analyses highlight the need for the quality control of soluble proteins prior to downstream purification steps. The chemical refolding of inclusion bodies could be considered an alternative to soluble expression; however, this process is labor-intensive and requires high capital costs. Therefore, soluble expression has always been favored, further emphasizing the importance of soluble protein quality control. Contrary to the commonly held belief that protein aggregation is an irreversible process, we demonstrated here that initial protein precipitates can be ‘solubilized’ by sonication using commonly accepted laboratory practices. The high molecular weight precipitates of oligomeric complexes were solubilized by physical splitting into small soluble aggregates (Figure 4).

What is the major cause of soluble aggregate formation? One possibility is that for multi-domain proteins, one domain is properly folded and thus highly soluble, whereas one or more remain unfolded or partially folded in an aggregation-prone state, causing the whole protein to form soluble aggregates like micelles [16]. Multi-domain proteins usually fold in a co-translational manner [27], where the folding of the N-terminal domain significantly affects the subsequent folding of downstream C-terminal domains [28]. The induction of recombinant protein expression is typically accelerated by over-expression; thus, the continual translation of a protein in high amount may exceed the folding capacity of the recombinant host, with any imbalance between translation and folding speeds resulting in incorrectly folded aggregates [1]. These problems associated with aggregation-prone proteins can be circumvented by fusion with a highly soluble carrier protein or co-expression with a chaperone [6,8]. These approaches often successfully increase the overall solubility of target proteins, but do not guarantee their functional quality, resulting in soluble, non-functional aggregates [16,18].

Various tools have become available for improving the yield of recombinant proteins [1,2,10,29]; however, most approaches still rely on routine sonication during the initial purification step, with solubility as initial criterion of protein quality. Accumulating evidence suggests that solubility is necessary, but not sufficient to determine biological function. Thus, screening out the non-functional fraction at the beginning of purification is necessary to control the quality of the proteins produced by recombinant technology. The same guidelines also apply to various approaches to circumvent inclusion body formation: fusion with highly soluble proteins [6,7,8,9,10,11], co-expression with molecular chaperones or specific binding partners to assist target protein folding, or harnessing the chaperna function of RNAs [12].

In conclusion, mild lysis is a useful method for screening out non-functional soluble aggregates during the early stages of purification to improve the overall quality of the protein of interest. The results of this study also highlight the importance of the folding environment in recombinant hosts on both the solubility and functional activity of proteins. Further progress and technical innovation is necessary to harness molecular chaperones [2,30] or chaperna [10,12,13,14] for improving the folding status of recombinant proteins. However, we believe that this study provides a rigorous evaluation of the folding quality of recombinant proteins, which may be conducive to the functional characterization of recombinant proteins for therapeutic, diagnostic, or prophylactic applications.

## 4. Materials and Methods

### 4.1. Construction of Protein Expression Plasmids

The EGFP gene was obtained by polymerase chain reaction (PCR) using specific primers. The PCR products were ligated into the pGE-hRID(2m) plasmid [14] via BamHI/HindIII sites. The secreted protein acidic and cysteine rich (SPARC) (GAA50260.1) gene was obtained by reverse transcription PCR (RT-PCR) from a crude lysate of *Clonorchis sinensis*, and the DNA fragments were ligated into the pGE-LysRS plasmid [10] via BamHI/SalI sites. The hexa-histidine tag for purification using Ni-affinity chromatography was located between the fusion partners and target genes.

### 4.2. Protein Expression and Cell Lysis

The expression plasmids pGE-hRID(2m)-6xhis-EGFP (hRID(2m)-EGFP) and pGE-LysRS-6xhis-SPARC (RS-SPARC), were transformed into the BL21(DE3)pLysE and BL21(DE3)pLysS *E. coli* strains, respectively. A single colony was inoculated into 3 mL Luria-Bertani (LB) medium containing 50 µg/mL ampicillin and 34 µg/mL chloramphenicol, then cultured overnight at 37 °C. Approximately 0.5 mL of the overnight cultured cells were transferred to 20 mL of fresh LB medium with the same concentration of antibiotics, then cultured at 37 °C until an optical density (OD)600 of 0.5 was reached. The expression of hRID(2m)-EGFP or RS-SPARC was then induced by adding 1 mM Isopropyl beat-D-1-thiogalactopyranoside (IPTG) followed by incubation for 3 h at 37 °C or 5 h at 27 °C. Approximately 10 mL of cultured cells were harvested and suspended in 0.3 mL of the mild lysis buffer B-PER (Thermo Scientific, 90078, Rockford, IL, USA). The suspended cells were sonicated or incubated at room temperature, and separated into total, soluble, and insoluble fractions by centrifugation at 15,000× *g* for 12 min at 4 °C. The expression of target proteins was analyzed by SDS-PAGE. The fluorescent protein bands and Coomassie Blue stained protein gels were imaged using a gel documentation system (InGenius3, SYNGENE, Frederick, MD, USA).

### 4.3. Protein Purification

A scale-up culture (0.5 L) was conducted for the purification of recombinant proteins using Ni-affinity chromatography. The harvested cells were suspended in 10 mL B-PER and sonicated as previously described [24], or mildly lysed in 10 mL B-PER, and then the two different methods were compared. Supernatants were obtained by centrifugation at 15,000× *g* for 12 min, and were loaded on to a nickel column equilibrated with A buffer [50 mL Tris-Cl (pH 7.5), 300 mM NaCl, 10% glycerol, 10 mM imidazole, 2 mM beta-mercaptoethanol, and 0.1% Tween-20]. After washing with the A buffer, target proteins were eluted with a concentration gradient of imidazole. Each fraction was analyzed by SDS-PAGE, and the fractions enriched with the target protein were pooled and dialyzed against the storage buffer [50 mM Tris-Cl (pH 7.5) and 300 mM NaCl].

For SEC, some of the fractions from the Ni-affinity chromatography were loaded on to a Superdex 200 Increase 10/300 GL column or a 10/300 Superose^TM^ 6 Increase column (GE Healthcare, Chicago, IL, USA). Before loading, the gel filtration column was equilibrated with two column volumes of buffer [50 mM Tris-Cl (pH 7.5) and 300 mM NaCl]. Proteins were loaded at 3% column volume, and eluted at a flow rate of 0.3 mL/min using a NGC^TM^ chromatography system (Bio-Rad Laboratories, Irvine, CA, USA).

### 4.4. Dynamic Light Scattering (DLS)

The harvested cells were lysed using the mild lysis method and divided into soluble and pelleted fractions by centrifugation at 15,000× *g* for 12 min. The pellet was suspended in PBS and transferred to four e-tubes. Each tube was sonicated for 0, 6, 30, or 72 s, and the size distribution was measured by DLS (ELS-1000ZS, Otsuka electronics, Osaka, Japan).

### 4.5. Fluorescence Measurement

The fluorescence of EGFP in crude lysates or after purification was measured using a microplate reader (FLUOstar OPTIMA, BMG LABTECH, Ortenberg, Germany). The measurements were carried out using a 96 well plate at RT (excitation wavelength: 488 nm and emission wavelength: 520 nm).
